# On the Similarity of Pulsating and Accelerating Turbulent Pipe Flows

**DOI:** 10.1007/s10494-017-9855-5

**Published:** 2017-09-20

**Authors:** L. R. Joel Sundstrom, Michel J. Cervantes

**Affiliations:** 1 Division of Fluid and Experimental Mechanics, Luleå University of Technology, SE-971 87 Luleå, Sweden; 2 Department of Energy and Process Engineering, Norwegian University of Science and Technology, NO-7491 Trondheim, Norway

**Keywords:** Pipe flow, Unsteadiness, Wall shear stress, Turbulence

## Abstract

The near-wall region of an unsteady turbulent pipe flow has been investigated experimentally using hot-film anemometry and two-component particle image velocimetry. The imposed unsteadiness has been pulsating, i.e., when a non-zero mean turbulent flow is perturbed by sinusoidal oscillations, and near-uniformly accelerating in which the mean flow ramped monotonically between two turbulent states. Previous studies of accelerating flows have shown that the time evolution between the two turbulent states occurs in three stages. The first stage is associated with a minimal response of the Reynolds shear stress and the ensemble-averaged mean flow evolves essentially akin to a laminar flow undergoing the same change in flow rate. During the second stage, the turbulence responds rapidly to the new flow conditions set by the acceleration and the laminar-like behavior rapidly disappears. During the final stage, the flow adapts to the conditions set by the final Reynolds number. In here, it is shown that the time-development of the ensemble-averaged wall shear stress and turbulence during the accelerating phase of a pulsating flow bears marked similarity to the first two stages of time-development exhibited by a near-uniformly accelerating flow. The stage-like time-development is observed even for a very low forcing frequency; $\omega ^{+}=\omega \nu /{\overline {u}}_{\tau }^{2}=0.00073$ (or equivalently, ${l}_{s}^{+}=\sqrt {2/\omega ^{+}}=52$), at an amplitude of pulsation of 0.5. Some previous studies have considered the flow to be quasi-steady at ${l}_{s}^{+}=52$; however, the forcing amplitude has been smaller in those studies. The importance of the forcing amplitude is reinforced by the time-development of the ensemble-averaged turbulence field. For, the near-wall response of the Reynolds stresses showed a dependence on the amplitude of pulsation. Thus, it appears to exist a need to seek alternative similarity parameters, taking the amplitude of pulsation into account, if the response of different flow quantities in a pulsating flow are to be classified correctly.

## Introduction

When a wall-bounded non-zero mean flow is perturbed by periodic, harmonically oscillating unsteadiness, it is termed a pulsating flow. Two motivations for studying pulsating flows are i) many flows in engineering applications such as that in turbomachinery are pulsatile, and thus, having a better understanding of such flows can lead to design improvements. ii) From a fundamental perspective, because the unsteadiness impose non-equilibrium features like phase shifts between turbulence production and dissipation that are not present in a steady-state flow.

If the flow is laminar, analytical solutions exist for pulsatile boundary-layer, channel and pipe flows (see [1, 2] and [3], respectively). For canonical turbulent wall-bounded flows, on the other hand, no analytical solutions exist. The common approach to analyze pulsating turbulent flows is to fit Fourier series to the ensemble-averaged mean and turbulent quantities, obtained from either measurements or simulations. To understand, or at least to classify, how the amplitude and phase of these Fourier series depend on the time-averaged bulk flow Reynolds number $Re=\overline {U}_{b}D/\nu $ and the angular forcing frequency *ω* = 2*π* *f*, similarity parameters to correlate the results must be sought. $\overline {U}_{b}$, *D*, *ν* and *f* denote the time-averaged bulk velocity, pipe diameter, kinematic viscosity and forcing frequency, respectively. A natural candidate as a starting point for scaling is the laminar Stokes length $l_{s}=\sqrt {2\nu /\omega }$ which, in both Stokes’ second problem (the oscillating plate) and in laminar pulsating flows, is a measure of how far the oscillating shear generated at the wall diffuses into the flow. The viscous length scale $\delta _{\nu } = \nu /\overline {u}_{\tau }$; with $\overline {u}_{\tau }$ being the time-averaged friction velocity, is commonly used for defining different spatial regions of steady wall-bounded turbulent flows. Scaling the Stokes length by the viscous length scale defines a Stokes-Reynolds number ${l}_{s}^{+}=l_{s}/\delta _{\nu }=l_{s} \overline {u}_{\tau }/\nu $, which is a measure of the level of interaction between the oscillating and mean flows. For small ${l}_{s}^{+}$ (< 8, say), the interaction is negligible leading to an effectively frozen response of the turbulence. As a consequence, the phase-averaged mean velocity distribution resembles a laminar profile. For large ${l}_{s}^{+}$ (> 18, say), significant interactions take place with wall-normal profiles of the amplitude of many oscillating quantities resembling their corresponding steady counterparts. Previous studies ([4–6], e.g.) have shown that many quantities do scale with the Stokes-Reynolds number (or equivalently the normalized circular frequency $\omega ^{+}=\omega \nu /{\overline {u}}_{\tau }^{2} = 2/{l}_{s}^{+2}$).

By definition, the Stokes length is a purely laminar concept. Therefore, to account for the increased momentum diffusivity in turbulent flows, Scotti and Piomelli [7] introduced a turbulent Stokes’ length. Based on an eddy viscosity hypothesis, they defined $l_{t}=\sqrt {2(\nu +\nu _{t})/\omega }$. $\nu _{t} = \kappa \overline {u}_{\tau } l_{t}$ and *κ* denotes the eddy viscosity and von Kármán constant, respectively. They showed that modulations of the ensemble-averaged mean velocity and turbulent fluctuations were confined within a distance of $2{l}_{t}^{+}$ (= 2*l*_*t*_/*δ*_*ν*_) from the wall, thus providing some justification for using ${l}_{t}^{+}$ as a scaling parameter.

Albeit quite successful correlations using either ${l}_{s}^{+}$ or ${l}_{t}^{+}$ have been obtained, it is reasonable to infer that by considering only the time-averaged value of *u*_*τ*_ much of the underlying dynamics of a pulsating turbulent flow are lost. Specifically, the direct numerical simulations (DNS) by He and Seddighi [8] of an accelerating channel flow undergoing a step change in flow rate, which is not a pulsating flow but still relevant, have shown that the ratio of initial to final Reynolds numbers characterizes the transient flow behavior. It is tempting to draw analogies between monotonically accelerating flows and the accelerating phase of pulsating flows and to postulate that the ratio of the maximum and minimum Reynolds numbers between which the flow oscillates (subscripted 1 and 0, respectively), should be a parameter of importance. The amplitude of oscillation $\widetilde {A} = (Re_{1} - Re_{0})/(Re_{1} + Re_{0})$, thereby defines another similarity parameter of potential importance. For $\widetilde {A}<1$, i.e., when the bulk flow does not reverse direction, former studies have, however, generally ascribed minor significance to the amplitude. Instead, as fore mentioned, the results have been scaled using only $\overline {u}_{\tau }$, *ω* and *ν* [5, 7, 9]. This approach may, under certain conditions such as for small imposed amplitudes, be sufficient if the only interest is to find a correlation for the response of the amplitude and phase of the fundamental mode of the aforementioned Fourier series function of $l_{s}^{+}$ (the fundamental mode have, in many studies, indeed contained a significant portion of the power-spectral density, see [10], e.g.). This approach does, however, neglect all details of the oscillating flow cycle.

Unfortunately, time-resolved data from pulsating flows are sparse, where the large-eddy simulation (LES) presented in [7], and the DNS by [11] are two exceptions. Comparing the turbulence response predicted by the LES during the accelerating phase of the oscillating cycle with the results from the DNS by [12] of a linearly accelerating flow, reveals striking similarities between the two cases. The initial response of the turbulence is in the streamwise fluctuating velocity in terms of streak amplification, and subsequently after an initial delay, the wall-normal and spanwise components increase rapidly in response to the formation and merging of turbulent spots. Qualitatively, this development of the turbulence in a pulsating flow can be inferred from the fundamental mode of the Fourier series by calculating the phase-difference between the ensemble-averaged turbulent fluctuations. Nonetheless, the relevant mechanisms underlying the flow response are unavoidably bypassed utilizing such approach.

By starting from the existing knowledge about how an accelerating flow develops in time, the purpose of the present paper is to reinforce the similarity between a monotonically accelerating turbulent flow, and the accelerating phase of a pulsating turbulent flow. To that end, hot-film measurements of the wall shear stress as well as two-component particle image velocimetry (PIV) measurements have been performed in a turbulent pipe flow subjected to either pulsed or transient unsteadiness. The PIV experiments were designed such that the flow rate histories were similar among the cases (i.e., the ramp time of the accelerating flow was close to half of the pulsation period, and the ratio of initial to final Reynolds numbers was approximately two times $\widetilde {A}$). This setup enables direct comparison between the flows, and as a direct consequence, the shortcoming of characterizing pulsating flows solely on the time-averaged friction velocity and the forcing frequency. Note that the proposed similarity between accelerating and pulsating flows is restricted to cases that do not involve relaminarization or flow reversal, since such conditions might change the flow development considerably.

The literature covering pulsating and transient flows is vast. In here, no attempt was made to summarize the topics. Readers interested in an introduction to pulsating flows are referred to either [6, 13] or [7]. More details on transient flows are found in [14] or [12], e.g.

## Measurement Methods and Data Reduction

### Experimental facility

The experimental facility consisted of a 10.4 m straight pipe having an internal diameter *D* = 2*R* = 100 mm. The working fluid was supplied to the test section through a piping system using an Oberdorfer N1100 gear pump. The bulk velocity, *U*_*b*_, was monitored using a Krohne OPTIFLUX electromagnetic flow meter having an accuracy at steady operational conditions of ± 0.7%.

The experimental program was subdivided into two separate measurement campaigns. In one series of measurements, a hot-film sensor was used to measure the wall shear stress. In the second series of measurements, PIV was used to measure the axial and radial velocities. The experimental test section was mainly manufactured in Plexiglas. The Plexiglas pipe does not pose problems when performing hot-film measurement using water as the working fluid. Hence, water kept at 20 °C ± 0.1 °C was used as the working fluid for the hot-film measurements.

Because of its large refractive index (*n* = 1.49), the Plexiglas pipe obstructs near-wall PIV measurements if water, having a refractive index of 1.33, is used as the working fluid. To enable near-wall measurements in a pipe, the refractive indices of the pipe wall and the working fluid ought be matched. One possibility is to mix water with a salt of high refractive index, e.g. ammonium thiocyanate (Borrero-Echeverry and Morrison [15]), or sodium iodide (Bai and Katz [16]). Such salt-water mixtures remedies the optical distortions, but they do also pose problems. Ammonium thiocyanate is highly corrosive and hazardous, whereas a solution of water and sodium iodide looses its transparency if it comes into contact with UV-light and (or) oxygen (Scholz, Reuter and Heitmann [17]).

Therefore, to avoid using these types of salts, other means by which to match the refractive indices were sought. Previously, in the pipe flow laser Doppler velocimetry study by den Toonder and Nieuwstadt [18], the optical measurement section of the pipe had been replaced with a thin foil of fluorinated ethylene propylene (FEP), which has a refractive index *n* ≈ 1.34. This setup enabled measurements down to a distance of 200 *μ* m from the wall, corresponding to approximately seven viscous units at *R* *e* = 24,600. The success with which those authors performed their measurements inspired the use of a similar approach in this work. To that end, a section of the Plexiglas pipe was substituted for a one meter long pipe made out of FEP. A relatively long section of FEP was used to not have the connection between the Plexiglas and the FEP directly next to the measurement section, although care had been taken in the manufacturing procedure to make the connection between the materials flush. To eliminate the optical distortions at the curved pipe surface, approximately 5% (by volume) of glycerine was added to water, and an index-matching box filled with the same water-glycerine solution was placed around the FEP tube. The viscosity of the resulting fluid (at 20 °C) was determined to *ν* = 1.14 × 10^−6^ m^2^ *s* ^−1^ using a glass capillary viscometer (Ostwald viscometer). This value agreed within 2% of the empirical formula presented in Cheng [20]. The tabulated value was, however, deemed more accurate than the one determined experimentally. Therefore, the tabulated value was used for calculations involving the kinematic viscosity.

### Experimental conditions

**Fig. 1:**
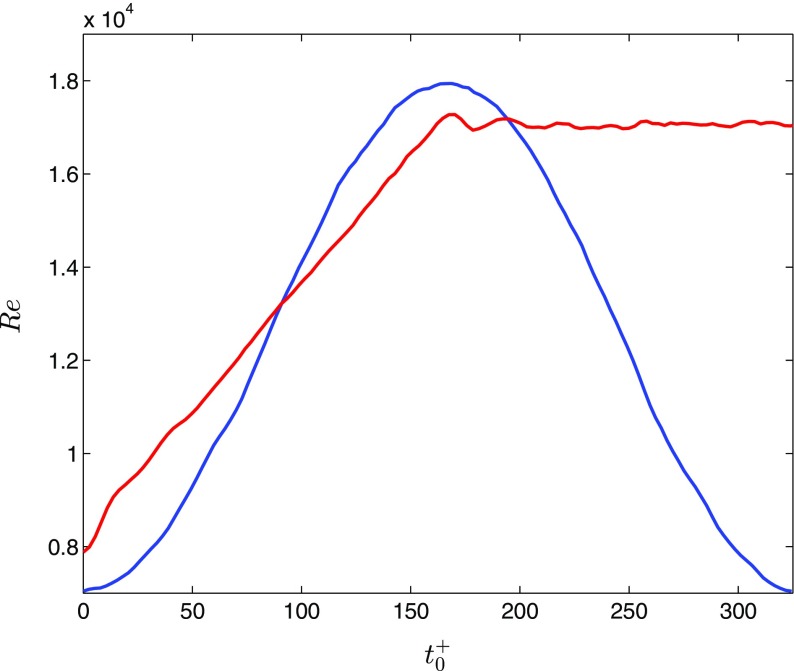
Reynolds number histories of test cases AP1 and PP1

Each measurement is characterized by the change in Reynolds number, Δ*R* *e* = *R* *e*_1_ − *R* *e*_0_ and the ramp time Δ*T* = *t*_1_ − *t*_0_. The indices 1 and 0 refer to the maximum and minimum values (for pulsating flows, the period time is twice Δ*T*). Table 1 summarizes the measurement cases to be discussed. Note that for the cases of accelerating flows, the change in the bulk flow rate is close to, but not strictly, linear. For the two cases termed AP1 and PP1, detailed comparisons of the evolution of the turbulence will be performed. It is therefore instructive to show the Reynolds number histories for these cases, see Fig. 1. Note that the data from PP1 (as well as PH1, PH2 and PH3) have been shifted such that the accelerating phase of the bulk flow starts at ${t}_{0}^{+} = {tu}_{\tau 0}^{2}/\nu =0$. *u*_*τ*0_ denotes the minimum friction velocity.

**Table 1:** Experimental conditions

Case	Re$_{0}$	Re$_{1}$	$\Delta T$ (s)	$\Delta T^{+}$	$\omega ^{+}$	${l}_{s}^{+}$	$\delta $	$\widetilde {A}$
AP1	7,900	17,100	6.1	170	–	–	0.007	–
PP1	7,100	18,000	6.25	162	0.008	16	–	0.43
AP2	7,900	17,100	3	85	–	–	0.013	–
PP2	10,500	14,300	6.25	162	0.008	16	–	0.15
AH1	11,400	35,700	7.25	355	–	–	0.0057	–
PH1	7,200	22,200	12.5	380	0.0031	25	–	0.5
PH2	7,200	22,200	25	680	0.0015	36	–	0.5
PH3	7,200	22,200	50	1,350	0.00073	52	–	0.5

The first letter, A or P, stands for accelerating and pulsating, respectively. The second letter, P or H, stands for the measurement method used, i.e., PIV or Hot-film. Indices 0 and 1 represent the minimum and maximum values (note that the period time for a pulsating flow is $2\Delta T$). The parameter $\delta = \frac {\nu }{{u}_{\tau 0}^{2}} \frac {1}{U_{b0}} \frac {\mathrm {d}U_{b}}{\mathrm {d}t}$, was used by [19] to correlate their data in near-uniformly accelerating flows

### Instrumentation

#### Particle image velocitmetry measurements

The axial (*U*) and radial/wall-normal (*V*) velocity components as defined in a standard cylindrical coordinate system were measured using a PIV system from Dantec Dynamics.

The laser sheet was formed by passing the beams of a double-pulsed Nd:YAG laser with a power of 50 mJ per pulse at 532 nm through a cylindrical lens. The particle images were captured using a 2048 by 2048 pixel camera at 10 Hz (the maximum frame rate of the camera). The velocity vectors were calculated using the commercially available software DynamicStudio from Dantec, using an adaptive cross correlation algorithm on pairs of single exposed images. Each image was subdivided into rectangular interrogation areas (IAs) of size 64 by 256 pixels in the vertical and horizontal directions, respectively. The IAs were elongated in the horizontal direction to enable measurements of the large range of velocities encountered during the experiments. To increase the wall-normal resolution, the IAs were overlapped by 50% in the vertical direction, resulting in 64 by 8 IAs in the vertical and horizontal directions, respectively. Because of inflow and outflow of particles between the two images, the data in the first two and the last two columns of the IAs were of low quality. These four columns were disregarded in the post-processing.

To detect spurious vectors, a 3 by 3 local median test was applied to the calculated velocity vectors (see Westerweel and Scarano [21]). The vectors not passing through this test were disregarded.

To facilitate a high wall-normal resolution close to the wall, the near-wall and bulk flow regions were measured separately. The near-wall field of view was 6.2 mm by 6.2 mm, resulting in a spatial resolution of 97 *μ* m by 390 *μ* m. This corresponded to a wall-normal resolution of 0.5 and 1 viscous units at the lowest and highest Reynolds numbers, respectively. The bulk flow was measured at a field of view of 50 mm by 50 mm; thereby giving a wall-normal resolution of 4 and 8 viscous units at the lowest and highest Reynolds numbers. Although the near-wall spatial resolution ranges between 0.5–1 viscous units, measurements of the wall-normal turbulent fluctuations below *y* ^+^ = 3 (based on the larger viscous scale) could not be achieved with satisfactory accuracy and will hence not be presented.

To minimize the disturbances resulting from the light scattered at the wall, a filter that effectively removed the light at the laser wave length from the PIV images was placed on the camera lens. Rhodamine B coated fluorescent Polymethyl methacrylate particles with maximum excitation/emission at 532 nm and 585 nm, respectively, were used for seeding. Calibration was performed by photographing a square plate equipped with a 125 *μ* m equispaced dot pattern. Owing to the index-matching, a one-dimensional calibration sufficed.

### Hot-film measurements

The wall shear stress was measured using a flush-mounted 55R46 hot-film sensor from Dantec. The sensor’s overheat ratio was set to 8%, using a Dantec Streamline Pro Constant Temperature Anemometry system. The sensing element of the probe measures 0.20 mm by 0.75 mm in the streamwise and circumferential directions, respectively. The dimensions in viscous units were below 15 *δ*_*ν*_ at the highest Reynolds number (35,500) encountered in the experiments.

Calibration of the sensor was performed in situ before and after each measurement series. The hot-film voltage and the flow rate were recorded at five Reynolds numbers. The lower Reynolds number was fixed at 5,000. The upper limit of the Reynolds number was 35,500 for cases PH1, PH2 and PH3, whereas it was 70,000 for case AH1. The wall shear stress at each point was estimated from $\tau =(\rho {{U}_{b}^{2}} f)/8$. The friction factor was extracted from the Colebrook formula, see [22]. The estimated values of the wall shear stresses were aptly fitted to the measured hot-film voltages, *E*, by the ‘standard’ relation *τ* ^1/3^ = *A* + *B* *E* ^2^. The hot-film signal was digitized using a PXI system consisting of a 24-bit NI-4472 card at a rate of 1 kHz.

### Data reduction

For the PIV data, ensemble averages were calculated by averaging over *N* = 250 repeated runs and *L* = 4 axial measurement points at the same phase of the flow rate excursions

1 $$\begin{array}{@{}rcl@{}} \langle\phi\rangle(r,t)&=&\frac{1}{NL}\sum\limits_{l=1}^{L}\sum\limits_{n=1}^{N} \phi_{n}(x_{l},r,t),\\ \langle\phi^{\prime}\phi^{\prime}\rangle(r,t)&=&\frac{1}{NL}\sum\limits_{l=1}^{L}\sum\limits_{n=1}^{N} \left[\phi_{n}(x_{l},r,t)-\langle\phi\rangle\right]^{2}. \end{array} $$

Where $\phi _{n}(x,r,t)=\langle \phi \rangle (r,t)+{\phi }_{n}^{\prime }(x,r,t)$ is the n:th measurement of a generic variable with ensemble average mean 〈*ϕ*〉(*r*, *t*), and deviation $\phi _{n}^{\prime }(x,r,t)$ from the ensemble-averaged mean. The averaging procedure for the hot-film data was similar, except that only one axial measurement position was available.

**Fig. 2:**
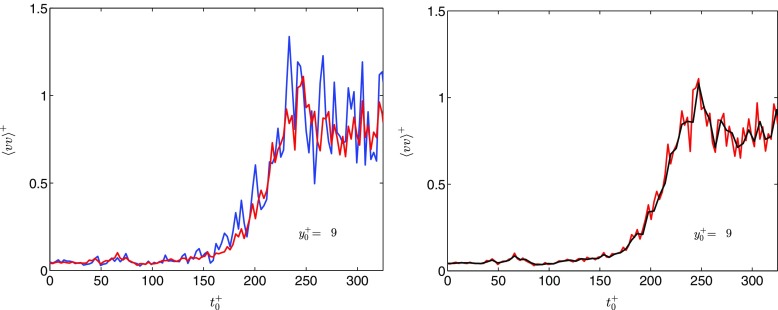
Check on the convergence of the data: , 50 repetitions; , 250 repetitions; , 250 repetitions with window averaging

Ideally, several thousands of repetitions have to be performed in order to achieve fully converged statistics. Performing this many repetitions is not feasible because of the large memory storage requirements for the PIV data. The number of repetitions performed (*N* = 250), was chosen as a compromise between the available data storage capacity and the convergence of the turbulence statistics. Figure 2a illustrates the convergence of the data by comparing the ensemble-averaged radial velocity fluctuations at a wall-normal distance ${y}_{0}^{+}=9$ for *N* = 50 and *N* = 250 repetitions. The overall trend is not affected by the number of repetitions, but, the fluctuations are reduced significantly for *N* = 250. However, even for the larger number of repetitions, non-negligible fluctuations are ubiquitous in the data. To dampen these fluctuations, the mean value of two consecutive time steps was calculated and used as a single time-step. The temporal resolution was thus reduced to 0.2 s. A similar approach has been used by [14, 19] in their studies of transient flows (they termed the approach window averaging). The averaging procedure also reminisces dividing the phase into equally spaced bins for calculating phase averages, as commonly done in studies of pulsating flows (see, e.g., [5]). Figure 2b shows that the window averaging approach reduces, but does not eliminate, the fluctuations compared to the raw data. Hence, the data to be presented in Section 3 will exhibit some fluctuations. The fluctuations are, however, not large enough to inhibit the discussion.

Similarly, the hot-film data will exhibit fluctuations despite the significant number of repetitions performed (at least 150). Owing to the higher sampling frequency, 100 consecutive data points (from each repetition) were stored in each window.

When the results are discussed, the ensemble-averaged (commonly also termed phase-averaged) mean velocity and wall shear stress will be termed *ensemble-averaged mean*, *phase-averaged mean* and *mean* interchangeably. The mean wall shear stress is presented with a lowercase *τ*, the ensemble-averaged mean velocity is presented with a capital *U*, and the ensemble-averaged Reynolds stresses are presented as 〈*u* *u*〉, 〈*v* *v*〉 and 〈*u* *v*〉. For all measurements, the mean radial velocity was at least two orders of magnitude smaller than *U*. Hence, only the fluctuating part of the radial velocity *v*, and its correlation with *u*, are presented.

## Results

The results section is divided into five parts. Initially, to verify the present experimental setup, time-averaged data are discussed in relation to well-known results. Comparisons are also made for the amplitude and the phase of the phase-averaged mean axial velocity. The second sub-section covers the time-development of the wall shear stress. This section is followed by a presentation of the Reynolds stresses and a section presenting further wall shear stress measurements. Finally, the influence of varying the amplitude of the imposed pulsation is elucidated by investigating the Reynolds stresses for $\widetilde {A}=0.15$ and $\widetilde {A}=0.43$.

The axial, radial and circumferential coordinates are denoted *x*, *r* and *θ*, respectively. The wall-normal distance *y* = *R* − *r*, scaled in viscous units *y* ^+^ = *y* *u*_*τ*_/*ν*, will be used frequently throughout the discussion. The time from the commencement of the acceleration scaled in viscous units, i.e., $t^{+}={tu}_{\tau }^{2}/\nu $, will also be used throughout the remainder of the paper. In Fig. 4, the wall-normal coordinate is scaled using the laminar Stokes length, i.e., *y*_*s*_ = *y*/*l*_*s*_.

### Characteristics of the time-averaged and phase-averaged fields

The current section serves as a verification of the adequacy of the present setup. Comparisons are made for the time-averaged mean axial velocity and turbulent fluctuations. Characteristics of the phase-averaged mean axial velocity are also discussed.

**Fig. 3:**
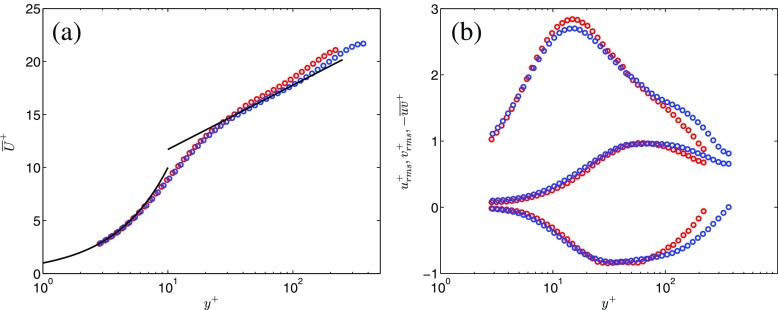
Time-averaged profiles of the axial velocity, the r.m.s. turbulent velocity fluctuations and the Reynolds shear stress scaled in viscous units. , AP1 before the commencement of the acceleration; , PP1

Figure 3a shows the time-averaged mean axial velocity before the commencement of the acceleration for case AP1 (*R* *e*_0_ = 7,900) and the time-averaged mean axial velocity for case PP1 ($\overline {Re}=12,550$). Close to the wall (*y* ^+^ < 5), the velocity profiles closely follow the law of the wall $\overline {U}^{+}=y^{+}$. Away from the wall (*y* ^+^ > 30), both cases display a logarithmic behavior but the velocity distributions do not collapse on the same curve. The profile corresponding to the lower Reynolds number (AP1) is slightly elevated in the ‘logarithmic layer’, this being a typical feature of low-Reynolds number flows [23]. From what Reynolds number, if any, there exists a universal logarithmic profile is debatable [24]. The Reynolds numbers investigated in here are for sure too low for a universal behavior of the time-averaged profiles to exist outside the buffer layer.

In Fig. 3b the root mean square (r.m.s.) turbulent fluctuations and the time-averaged Reynolds shear stress are plotted. The curves collapse quite closely for *y* ^+^ < 40. The peak of $u^{+}_{rms}$ is 2.85 and 2.7 for case AP1 and PP1, respectively. The location of the peak occurs at the typical wall-normal position *y* ^+^ ≈ 15. The difference (5%) in the peak values is likely related to measurement errors, taking into consideration the uncertainties involved in i) measuring the maximum value of *u*_*rms*_, and ii) estimating the friction velocity.

**Fig. 4:**
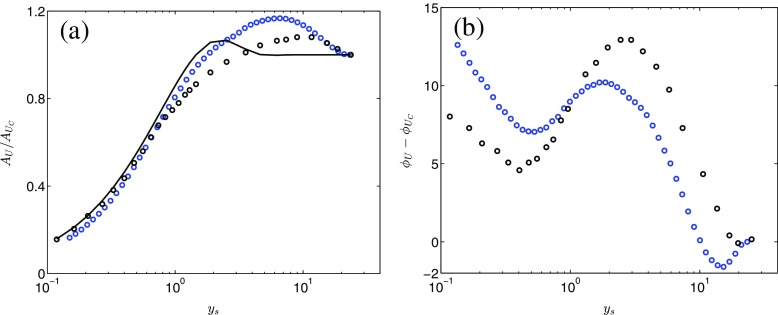
Amplitude and phase of the phase-averaged axial velocity. , PP1; ∘, data from Weng et al. [11]

Figure 4a, b shows, respectively, the amplitude and the phase of the phase-averaged mean axial velocity from case PP1 plotted versus *y*_*s*_ = *y*/*l*_*s*_. The data is compared with the channel flow DNS of Weng et al. [11] (${l}_{s}^{+}=18$). Both profiles do show fairly good agreement with the reference data; complete similarity cannot be expected because of the differences in ${l}_{s}^{+}$ and in the geometry. The phase difference displays an interesting behavior exhibiting a local minimum and a local maximum at *y*_*s*_ ≈ 0.5 and *y*_*s*_ ≈ 2, respectively. The reference data show a similar pattern but the wall-normal locations of the extrema are slightly different. The local maximum around *y*_*s*_ = 2 have been reported in many previous studies, [6, 25, 26], e.g. The minima, on the other hand, has not been reported as often. The cause, and potential effect of the peaks has not been given much attention in previous studies, though. It would be of interest to perform such study, however, that is outside the scope of the present paper.

### Ensemble-averaged mean and turbulent wall shear stress

#### Accelerating flow

**Fig. 5:**
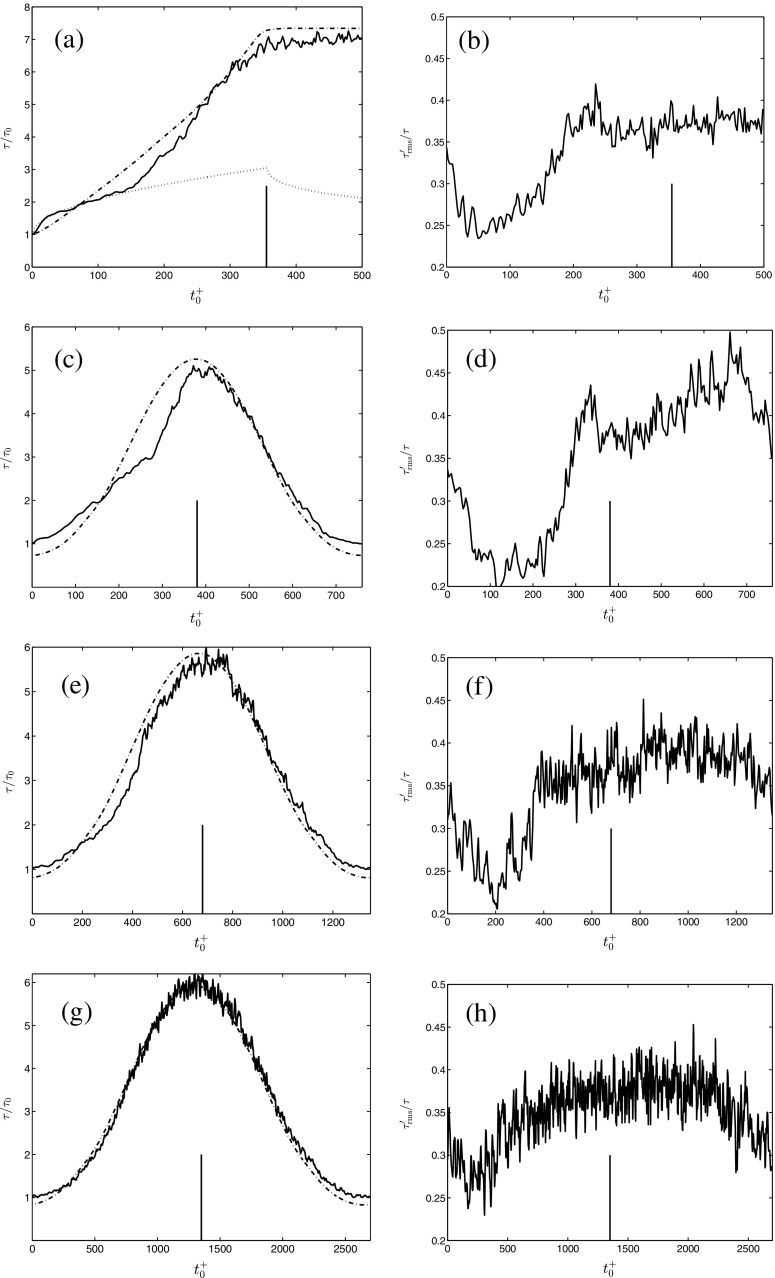
Time-development of: ensemble-averaged mean wall shear stress, left column of figures; ensemble-averaged r.m.s. turbulent wall shear stress, right column of figures. **a**–**b** AH1, *δ* = 0.0057; **c**–**d** PH1, ${l}_{s}^{+}=25$; **e**–**f** PH2, ${l}_{s}^{+}=36$; **g**–**h** PH3 ${l}_{s}^{+}=52$. , unsteady, , quasi-steady; , analytical laminar. The vertical bars denote the end of the bulk flow accelerating phase

**Fig. 6:**
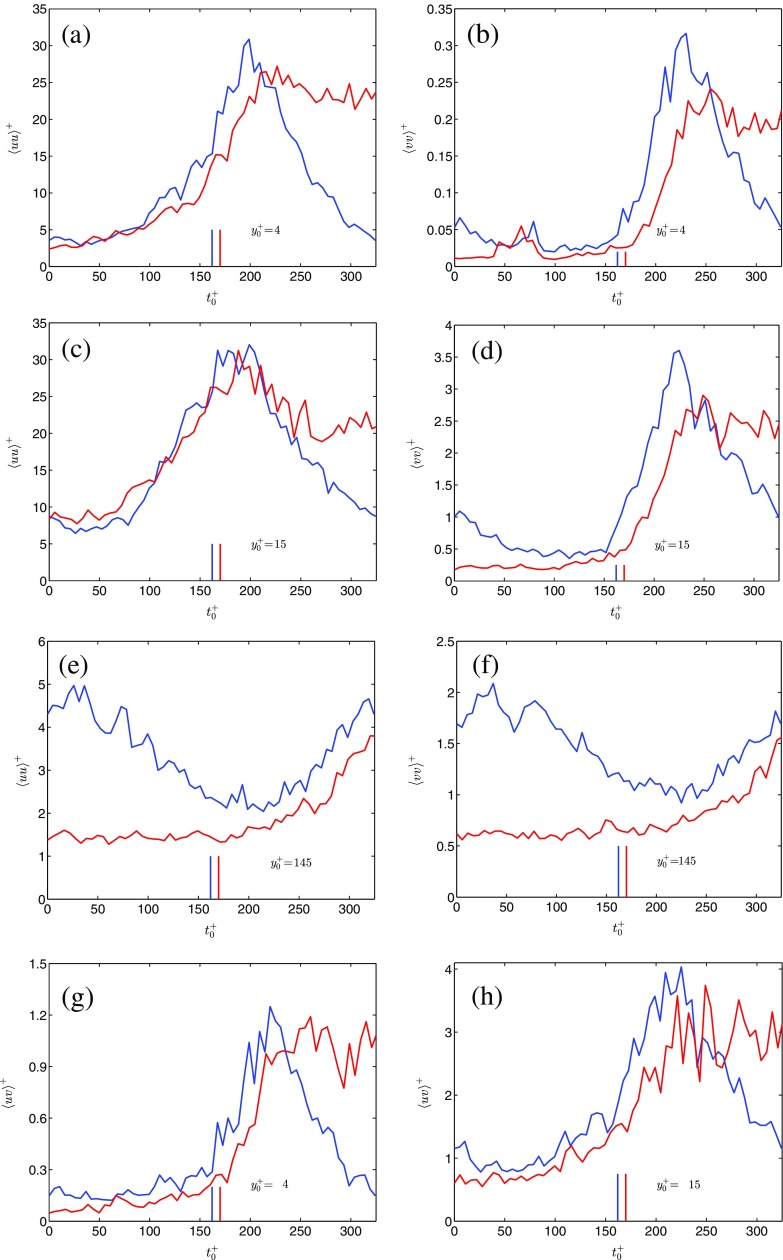
Time-developments of the ensemble-averaged Reynolds stresses. , PP1; , AP1. The vertical bars denote the end of the bulk flow accelerating phase

The time-development of the mean wall shear stress for case AH1 is shown in Fig. 5a. On the abscissa, time is scaled using the minimum friction velocity and the kinematic viscosity. The ordinate is scaled by the minimum wall shear stress. As shown previously by [19] and [12], and reconfirmed in here, the wall shear stress develops in three stages when the bulk flow undergoes a linear acceleration. Stage one is identified between $0<{t}_{0}^{+}<150$, stage two between $150<{t}_{0}^{+}<300$, and finally, stage three ${t}_{0}^{+}>300$. Initially, during the first stage, inertia dominates the response and *τ* increases from the value prevailing before the commencement of the acceleration akin to a laminar flow undergoing the same flow rate excursion (the response for a purely laminar flow is plotted as the dotted line in Fig. 5a, consult [27] for the derivation). Furthermore, the initial response of the wall shear stress is more rapid than in the corresponding steady-state flow, *τ*_*qs*_. The quasi-steady wall shear stress was obtained by calculating the (steady) friction factor at the instantaneous Reynolds number and subsequently using $\tau _{qs}=(\rho f {{U}_{b}^{2}})/8$. The close-to-laminar response of the flow is a result of delays in the time-development of the Reynolds shear stress following the commencement of the acceleration (the red lines in Fig. 6g, h, ${t}_{0}^{+}<100$, shows an example of this delay although the response of 〈*u* *v*〉 in that figure is from case AP1). As the first stage proceeds, however, the delayed response of the turbulence counteracts the effect of inertia, thus causing the transient *τ* to become smaller than the steady value (${t}_{0}^{+}>75$). During stage one, although the Reynolds shear stress remains approximately constant, there is a significant generation of streamwise turbulent velocity fluctuations. The generation of 〈*u* *u*〉 starts at the wall at ${t}_{0}^{+}=0$, and progressively propagates toward the pipe centreline as time proceeds, see Fig. 6a, c, e; although as for 〈*u* *v*〉, this is from a different case. As shown in the accelerating channel flow DNS by [12], the growth of 〈*u* *u*〉 during stage one is mainly associated with the excess shear that is generated at the wall as the flow accelerates. The excess shear elongates and amplifies the streamwise turbulence structures that pre-existed before the commencement of the acceleration. The growth of 〈*u* *u*〉 is therefore not associated with ‘normal’ turbulence activities that would exist in a statistically steady flow at the same Reynolds number (the elongated and amplified structures are akin to the growth of the streamwise disturbances in the buffeted laminar region in the laminar to turbulent boundary layer bypass transition). This anomaly development of the turbulence is what ultimately causes 〈*u* *v*〉 to remain approximately constant during stage one (and hence, the reason why the wall shear stress responds as in a laminar flow).

At ${t}_{0}^{+}=150$ the transient *τ* starts departing from the laminar solution, thus indicating that the turbulence is responding more rapidly to the imposed acceleration. The departure from the laminar solution signifies that the flow-development has entered the second stage. The generation of turbulence causes the wall shear stress to increase rapidly, and at ${t}_{0}^{+}=250$, *τ* has reached the same value as *τ*_*qs*_. Following the rapid increase, *τ* settles toward the value dictated by the final Reynolds number; i.e., the flow-development has progressed to the third stage. In this section, the response of the wall shear stress for a single case of accelerating flow is discussed. For a more thorough discussion on the response of the wall shear stress in accelerating pipe flows, including the influence of varying Δ*T*, Δ*R* *e* and *R* *e*_0_, the reader is referred to [19].

The response of the turbulence can also be illustrated by plotting the ensemble-averaged r.m.s. turbulent wall shear stress normalized by the mean wall shear stress. For steady-state flows, this quantity falls in the range $0.35<{\tau }_{rms}^{\prime }/\tau <0.40$ [28, 29]. Departure from these values is a good indicator of the flow being in a state of non-equilibrium. Figure 5b shows that ${\tau }_{rms}^{\prime }/\tau $ decreases rapidly following the commencement of the acceleration, reaching a value of 0.25 at ${t}_{0}^{+}=30$. Thereafter it remains approximately constant until ${t}_{0}^{+}=100$. The approximate constancy of the normalized r.m.s. wall shear implies that the growth of ${\tau }_{rms}^{\prime }$ is directly proportional to *τ*; i.e., that the growth of the streamwise velocity fluctuations is mainly associated with the excess shear generated at the wall, and not with a generation of Reynolds shear stress. Following this phase of approximately constant ${\tau }_{rms}^{\prime }/\tau $, the normalized r.m.s. wall shear stress increases steadily for $100<t_{0}^{+}<150$. This phase of slow growth of ${\tau }_{rms}^{\prime }/\tau $ implies that there is a generation of Reynolds shear stress during this period of time, albeit at a low rate. Then, for ${t}_{0}^{+}>150$ as the second stage starts, the rapid generation of turbulence causes the normalized r.m.s. value to increase rapidly, reaching a value of 0.385 at ${t}_{0}^{+}=200$. The normalized r.m.s. wall shear subsequently settle around 0.38 as the near-wall flow asymptotically reaches equilibrium.

#### Pulsating flow

Figure 5c, e, g shows, respectively, the mean wall shear stress for ${l}_{s}^{+}=25$, 36, and 52 for an amplitude of pulsation, $\widetilde {A}=0.5$. Although the accelerating phase of these flows start in a state of statistical non-equilibrium (as opposed to case AH1), much of the characteristics of the time-development of *τ* bears similarities to the stage-like development discussed previously.

Consider case PH1 (${l}_{s}^{+}=25$, Fig. 5c). Following the onset of the accelerating phase, the mean wall shear stress increases more rapidly than in the corresponding quasi-steady flow and overshoots these values (although part of the overshoot can be attributed to the fact that the minimum of the unsteady *τ* does not reach as low as the minimum quasi-steady wall shear stress). As the accelerating phase proceeds, however, the quasi-steady wall shear stress increases more rapidly than its unsteady counterpart and at ${t}_{0}^{+}=190$, *τ*_*qs*_ becomes larger than *τ*. At ${t}_{0}^{+}=270$, *τ* starts to increase rapidly and reaches the steady value at the end of the accelerating phase (${t}_{0}^{+}=375$). Clearly, there are similarities in the time-development of *τ* between cases PH1 and AH1. For case PH1 it can be thus inferred that the initial overshoot of *τ* relative to *τ*_*qs*_ is related to inertia, the subsequent undershoot to delays in the response of the turbulence, and finally, that the rapid increase of *τ* toward *τ*_*qs*_ stems from a rapid generation of turbulence.

The normalized r.m.s. turbulent wall shear stress further illustrates the similarity between accelerating and pulsating flows (see Fig. 5d). Following the commencement of the accelerating phase, ${\tau }_{rms}^{\prime }/\tau $ decreases rapidly to 0.25, and subsequently remains approximately constant for 170 time units. The duration of the phase of approximately constant ${\tau }_{rms}^{\prime }/\tau $ is longer for PH1 than for AH1. This is an effect of the smaller friction velocity at the commencement of the acceleration for PH1 than for case AH1. As for an accelerating flow, the constancy of ${\tau }_{rms}^{\prime }/\tau $ shows that ${\tau }_{rms}^{\prime }$ is directly proportional to *τ*; i.e., that the growth of 〈*u* *u*〉 is related to elongation and amplification of the streaks that exist at the commencement of the accelerating phase, and not to a generation of new turbulence structures (this can be seen in Fig. 6g, showing the time-development of Reynolds shear stress for case PP1). At ${t}_{0}^{+}=260$ the normalized r.m.s. wall shear stress increases rapidly in conjunction to the generation of new turbulence structures.

A pulsating flow starts to decelerate directly following the accelerating phase. Therefore, the third stage that is observed for an accelerating flow, i.e. when the flow approaches equilibrium, is never reached for a pulsating flow. In passing, it is interesting to note that *τ* overlaps with *τ*_*qs*_, during a significant portion of the decelerating phase. Hence, from a computational perspective, it should be easier to model the decelerating phase. It is, however, beyond the scope of this paper to investigate the decelerating phase, or any modeling issues, in greater detail.

Similarly, for case PH2 (${l}_{s}^{+}=36$), *τ* is seen to develop in stages showing the importance of, respectively, inertia, a delayed turbulence response and the subsequent rapid generation of turbulence (see Fig. 5e). However, because of the longer period time of the pulsation, the effects of the unsteadiness are considerably weaker than in case PH1. Even for case PH3, in which ${l}_{s}^{+}=52$, the effects of the flow unsteadiness can be traced in the time-developments of *τ* and ${\tau }_{rms}^{\prime }/\tau $ (Fig. 5g, h). The unsteadiness seen in the time-development for ${l}_{s}^{+}=52$ could be inferred as somewhat controversial since, e.g., [11] classified ${l}_{s}^{+}=45$ as quasi-steady. The imposed amplitude in that study was only 0.1. To reconcile the apparent conflict, it can be noted that [7] pointed out that the exact limit of quasi-steadiness is likely to be dependent on the amplitude of pulsation.

### Ensemble-averaged Reynolds stresses

Figure 6 shows the three measured components of the ensemble-averaged Reynolds stresses at wall-normal locations $4<{y}_{0}^{+}=yu_{\tau 0}/\nu <145$. The abscissa is scaled as in Fig. 5, and the ordinate is scaled using the minimum friction velocity.

Figure 6a shows the response of the streamwise component from cases AP1 and PP1 at ${y}_{0}^{+}=4$. For case AP1, 〈*u* *u*〉 increases at a slow but definite rate for ${t}_{0}^{+}<150$. As shown in the DNS by [12], and as discussed in Section 3.2.1, this initial increase is associated with an amplification and elongation of the near-wall streaks in relation to the excess shear generated at the commencement of the acceleration. At this phase there is hardly any generation of new turbulence structures, this being nicely illustrated in Fig. 6d (*t* ^+^ < 150) by 〈*v* *v*〉, which remains largely unchanged. For $150<{t}_{0}^{+}<210$, 〈*u* *u*〉 increases rapidly owing to generation of Reynolds shear stress (see Fig. 6g, ${t}_{0}^{+}\approx 150$). A significant portion of this increase occurs after ${t}_{0}^{+}=170$, i.e., when the bulk flow has stopped accelerating. Finally for ${t}_{0}^{+}>200$, 〈*u* *u*〉 settles toward the equilibrium value dictated by the final Reynolds number. For case PP1, 〈*u* *u*〉 exhibits a slow increase between $0<{t}_{0}^{+}<150$. This increase is readily associated with the excess shear that is generated at ${t}_{0}^{+}=0$. Subsequently, for $150<{t}_{0}^{+}<200$, 〈*u* *u*〉 increases rapidly. The rapid increase largely overlaps with a fast response of the Reynolds shear stress (Fig. 6g, ${t}_{0}^{+}\approx 150$ ). As for case AP1, a significant portion of the rapid increase of 〈*u* *u*〉 occurs when the bulk flow has stopped accelerating ($t_{0}^{+}=162$). Directly after 〈*u* *u*〉 has reached its peak value, the decaying phase starts because of the bulk flow deceleration. Clearly, as for the wall shear stress, the turbulent fluctuating streamwise velocity in a pulsating flow exhibits two of the three stages of time-development described in Section 3.2.

Further out in the near-wall region (Fig. 6c, ${y}_{0}^{+}=15$), 〈*u* *u*〉 still develops similarly between the two cases. Specifically, 〈*u* *u*〉 remains largely constant for ${t}_{0}^{+}<25$, increases slowly during the subsequent 50 time units, and then increases much more rapidly for $75<{t}_{0}^{+}<175$. For case AP1 it is interesting to note that 〈*u* *u*〉 decreases by more than 33% from its maximum value between $200<{t}_{0}^{+}<250$ and overlaps with 〈*u* *u*〉 from case PP1 until ${t}_{0}^{+}\approx 250$.

In the bulk flow (${y}_{0}^{+}=145$), there is a delay in the response of 〈*u* *u*〉 of approximately 200 time units for AP1, see Fig. 6e. Similarly, for PP1, 〈*u* *u*〉 passes through its minimum around ${t}_{0}^{+}\approx 200$, and subsequently starts increasing. Thus, the disturbance growth that is induced in the streamwise component at ${t}_{0}^{+}=0$ propagates away from the wall at nearly the same speed for the two cases. At this wall-normal position, the radial component develops similarly as the streamwise component, see Fig. 6f. The similarity in the time-development between 〈*u* *u*〉 and 〈*v* *v*〉 at this wall-normal position, as opposed to the near-wall behavior, shows that turbulence diffusion is the main energy providing mechanism in the outer regions of the flow (see [14], for a further discussion).

Figure 6d shows the time-development of 〈*v* *v*〉 at ${y}_{0}^{+}=15$. For case AP1, 〈*v* *v*〉 remains largely unchanged from the value prevailing before the commencement of the acceleration when ${t}_{0}^{+}<100$. Since 〈*v* *v*〉 does not extract energy directly from the mean flow, the low activity in 〈*v* *v*〉 during this period in time is a result of delays in the energy redistribution from 〈*u* *u*〉 by pressure-strain (the low activity of the pressure-strain is equivalent to the lack of generation of new turbulence structures, as discussed previously). During the subsequent 75 time units 〈*v* *v*〉 grows slowly, showing that the pressure-strain has started to respond to the new flow conditions, although at a low rate. For $175<{t}_{0}^{+}<250$, 〈*v* *v*〉 increases very rapidly, thus showing that the pressure-strain has become fully active and that new turbulence structures are generated. Subsequently, the wall-normal velocity fluctuations settle toward the final value. For PP1, 〈*v* *v*〉 decreases slightly between $0<{t}_{0}^{+}<90$, remain approximately constant for the next 60 time units and increases rapidly for $150<{t}_{0}^{+}<225$. Thus, due to the similarities to case AP1, the time-development of 〈*v* *v*〉 in a pulsating flow is also largely controlled by the response of the pressure-strain.

The time-development of 〈*v* *v*〉 next to the wall (${y}_{0}^{+}=4$, Fig. 6b) shows a peculiar behavior. For $40<{t}_{0}^{+}<90$ in the accelerating flow, and for $55<{t}_{0}^{+}<90$ in the pulsating flow, there is an increase, followed by a decrease of 〈*v* *v*〉. Note that the peculiarity disappears for ${y}_{0}^{+}>10$. Such peculiar behavior has not been found in DNS; neither in a linearly accelerating flow [12], nor in an impulsively accelerating flow [30]. However, large intermittency was reported during the first stage, see [12]. Hence, the peculiar behavior seen in the measurements might be a local asymmetry, thus showing up as intermittency in a DNS (in the evaluation of DNS data, homogeneity is assumed in the streamwise and circumferential/spanwise directions such that any local behavior will be smeared out in the averaging procedure). PIV measurements were performed in a single plane only. Hence, the peculiar behavior of 〈*v* *v*〉 could not be further investigated. However, asymmetries in the time-development of the wall shear stress have been found and these are discussed in the next section.

### Further wall shear stress observations

**Fig. 7:**
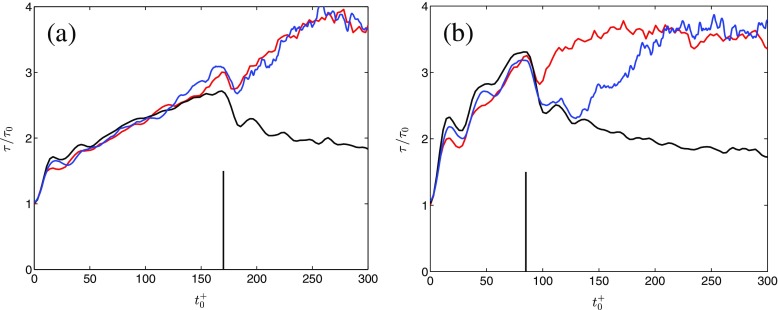
Time-developments of the ensemble-averaged mean wall shear stresses for **a** case AP1; **b** case AP2, computed from: , PIV; , Hot-film;  analytical laminar solution [27]. The vertical bars denote the end of the bulk flow accelerating phase

Wall shear stress measurements using near-wall data from PIV and a hot-film sensor were performed with a 90° circumferential separation. Note that this set of hot-film measurements were performed in the water-glycerine solution, and not in pure water as described in Section 2. Except for the difference in the fluids, the measurement approach for the hot-film was equivalent to that described in Section 2. Figure 7a shows the time-development of the ensemble-averaged mean wall shear stress from case AP1 alongside the aforementioned analytical laminar solution (the derivation is presented in [27] and [31]). Although some differences between the PIV and hot-film results are seen, it is difficult to discern if this is related to measurement errors or a circumferential effect.

The ensemble-averaged mean wall shear stress from case AP2 is plotted in Fig. 7b. As long as the acceleration is effective, there is good agreement between the measured wall shear stresses. Furthermore, the analytical (laminar) solution provides a fairly good representation of the data. Following the completion of the acceleration at ${t}_{0}^{+}=85$, the hot-film and analytical data start departing from the PIV data. Specifically, at the PIV station, the flow enters the second stage at ${t}_{0}^{+}=100$, whereas it remains in the first stage at the hot-film station until ${t}_{0}^{+}=130$. Thus, case AP2 exhibit significant circumferential dependence which, furthermore, is repeatable since the ensemble-averaged values differ depending on *θ*. Circumferential (and axial) dependence was also found by [19] in their hot-film measurements of *τ* in an accelerating pipe flow. The asymmetries were only pronounced in one out of eight cases; nonetheless, they showed that the time-development of the turbulence in a near-uniformly accelerating pipe flow may have a circumferential (and axial) dependence. How, or if, this circumferential dependence of *τ* is related to the peculiar time-development of 〈*v* *v*〉 seen in Fig. 6b for $40<{t}_{0}^{+}<90$ is unclear, and need further studies and data to be elucidated.

Before concluding this section, the following is worth noting. The production of 〈*u* *v*〉 is $\mathcal {P}_{\langle uv\rangle }=-\langle vv\rangle \partial U/\partial r$. The ensemble-averaged velocity is governed by the unsteady Reynolds-averaged Navier-Stokes equation which, for a fully developed axisymmetric flow, differ from a laminar equation only through the appearance of the Reynolds shear stress 〈*u* *v*〉. For case AP2 the laminar formulation of the wall shear stress is valid for different durations of time, this being a function of the circumferential coordinate. Thus, 〈*u* *v*〉 and ultimately 〈*v* *v*〉 as realized through the production $\mathcal {P}_{\langle uv\rangle }$, is a function of the circumferential coordinate for case AP2.

### Influence of forcing amplitude

**Fig. 8:**
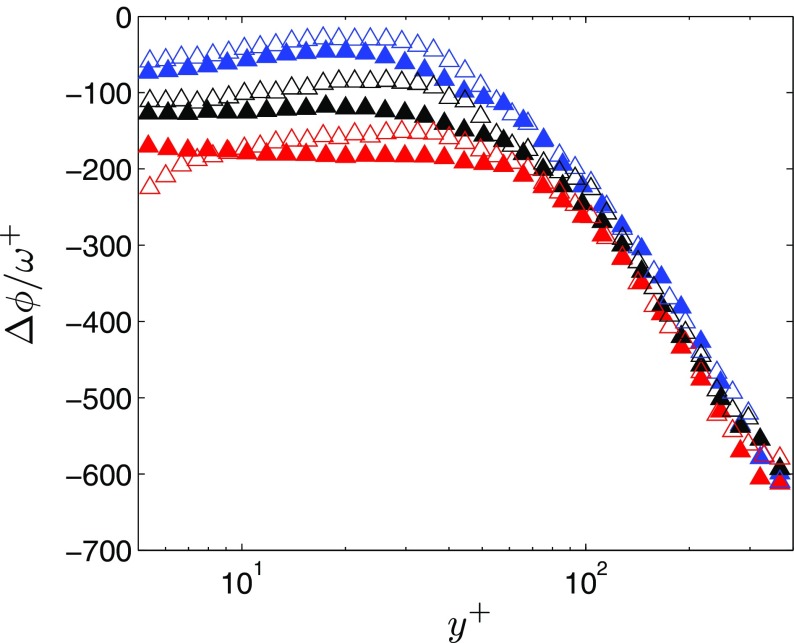
Time-lag relative to the centreline velocity oscillation for , 〈*uu*〉; , 〈*vv*〉; and Δ, 〈*uv*〉. Closed symbols, PP1; open symbols, PP2

In [19] it was shown that the delay time before the near-wall turbulence responded to a close-to-uniform acceleration was dependent on the initial Reynolds number, which defines a time scale $t_{\nu _{0}}=\nu /{u}_{\tau 0}^{2}$, that characterizes the turbulence. In here $t_{\nu _{0}}$ is 36 ms, 38 ms and 20 ms for AP1, PP1 and PP2, respectively. The similarity with which the turbulence evolves between cases AP1 and PP1 presented in Section 3.3 suggest that it is the minimum value of *u*_*τ*_ that defines the turbulent time scale (of the accelerating phase) in a pulsating flow. To reinforce the importance of *u*_*τ*0_, additional measurements were undertaken in a pulsating flow with the same time-averaged *u*_*τ*_, but with a larger *u*_*τ*0_ (case PP2 in Table 1). Figure 8 shows the time-lag in viscous units between the Reynolds stresses and the centreline velocity for PP1 and PP2. The time-lags have been calculated from the fundamental mode of the Fourier series of the respective flow quantity. The figure shows that for case PP2, which exhibits a larger *u*_*τ*0_, there are shorter time-lags of the turbulence relative to the centreline for *y* ^+^ < 60 (*y* ^+^ is derived based on the time-averaged friction velocity, not *u*_*τ*0_). Furthermore, the wall-normal distance at which the time-lag of 〈*v* *v*〉 starts to deviate from an approximately constant value (*y* ^+^ = 45 for PP2 and *y* ^+^ = 60 for PP1) is smaller for PP2; in conclusion, the time-lags do not scale with the same friction velocity.

The present results show that the forcing amplitude is an important parameter for the response of a pulsating flow. We do, however, refrain from claiming that similarity parameters such as ${l}_{s}^{+}$ and *ω* ^+^ (which are independent of the amplitude) lack meaning. For example, classifying the flow into different regimes, such as quasi-laminar, intermediate or quasi-steady, can be quite satisfactorily addressed by ${l}_{s}^{+}$ and *ω* ^+^. However, it cannot be expected that the response of different flow quantities will scale universally upon a similarity parameter that does not take the forcing amplitude into account. In here, no attempt was made to find new similarity parameters. Finding a new similarity parameter requires data from more cases covering different amplitudes, forcing frequencies and time-averaged Reynolds numbers.

## Conclusions

Two-component particle image velocimetry measurements and hot-film measurements of the wall shear stress in both pulsating and near-uniformly accelerating turbulent pipe flows have been presented. Statistics have been obtained by performing many repetitions of nominally similar flow rate excursions, thus allowing both ensemble-averaged mean and turbulent fluctuations to be calculated.

The main finding of this work is that the responses of both the wall shear stress and the turbulence are similar between a near-uniformly accelerating flow, and the accelerating phase of a pulsating flow. Previous studies, [19] and [12] e.g., have shown that a flow undergoing a near-uniform change in the bulk flow rate evolves in three distinct stages. The first stage is characterized by a dominance of inertia and minimal response of the turbulence (through the Reynolds shear stress, the streamwise fluctuating velocity does increase). The onset of the second stage is identified by a rapid generation of new turbulence, whereas in the last stage, the flow attains distributions of the mean and turbulence quantities as dictated by the final Reynolds number.

The response of the wall shear stress for one case of accelerating flow and three cases of pulsating flows was presented first. The three-stage development was reconfirmed for the accelerating flow. Two of the three stages were observed for pulsating flows over a large range of non-dimensional frequencies $0.00073<\omega ^{+}=\omega \nu /{\overline {u}}_{\tau }^{2}<0.0031$, or equivalently, $25<{l}_{s}^{+}<52$. The stage-like development was more distinct for the larger forcing frequency, but nonetheless, even for the lowest investigated frequency, non-negligible effects of the flow unsteadiness were observed. The pulsation amplitude was 0.5, which is moderately large. The stage-like time-development for ${l}_{s}^{+}=52$ is interesting because it illuminates the importance of taking the forcing amplitude into account for classifying a pulsating flow correctly. For example, [11] considered ${l}_{s}^{+}=45$ as quasi-steady; however, their forcing amplitude was only 0.1.

Subsequently, the response of the Reynolds stresses was presented. Specifically, as shown in previous studies, the initial response of the turbulence is through an increase of the near-wall streamwise fluctuating velocity in conjunction to the excess shear that is generated when the flow starts to accelerate. The Reynolds shear stress, and the wall-normal fluctuating velocity, on the other hand, remain largely unchanged or decreases slightly during the first stage. In the second stage there is a rapid generation of both 〈*u* *v*〉 and 〈*v* *v*〉.

During the first stage, there is a peculiar behavior in the wall-normal fluctuating velocity next to the wall (${y}_{0}^{+}<10$). For a brief period of time, 〈*v* *v*〉 increases, but subsequently decreases back to the same value. The phenomenon is observed for both types of flows, but a similar phenomenon has not been reported in previous studies. It is therefore conjectured that that the phenomenon is local in space, and furthermore repeatable, since it is observed for an ensemble-averaged quantity. The existence of an asymmetric response of the turbulence is supported by further wall shear stress measurements that were performed with a 90° circumferential separation. For, these measurements displayed a circumferential dependence. Such behavior of the wall shear stress, on the other hand, has been reported previously by [19].

Finally, it was shown that the time-delay of the ensemble-averaged Reynolds normal and shear stresses relative to the centreline velocity, is dependent on the forcing amplitude. This finding further highlights the importance of the amplitude of pulsation for understanding the physical processes underlying a pulsating flow. Previous studies ([4, 5, 7], e.g.) have ascribed only minor importance to the amplitude though. These studies have, generally, sought to correlate the response of turbulent and mean flow quantities based on similarity parameters that do not take the amplitude into account; i.e., *ω* ^+^ and ${l}_{s}^{+}$. Although the present results do stress the importance of the amplitude of pulsation, we refrained from seeking new similarity parameters because sufficient data was not captured to accomplish such task.
